# Thyroid Function Changes and Pubertal Progress in Females: A Longitudinal Study in Iodine-Sufficient Areas of East China

**DOI:** 10.3389/fendo.2021.653680

**Published:** 2021-05-11

**Authors:** Yingying Wang, Dandan He, Chaowei Fu, Xiaolian Dong, Feng Jiang, Meifang Su, Qian Xu, Peixin Huang, Na Wang, Yue Chen, Qingwu Jiang

**Affiliations:** ^1^ Department of Epidemiology, School of Public Health, Fudan University, Shanghai, China; ^2^ Key Laboratory of Public Health Safety of Ministry of Education, School of Public Health, Fudan University, Shanghai, China; ^3^ Department of School Hygiene, Minhang District Center for Disease Control and Prevention, Shanghai, China; ^4^ Department of Chronic Disease Control and Prevention, Deqing County Center for Disease Control and Prevention, Huzhou, China; ^5^ Department of Chronic Disease Control and Prevention, Yuhuan City Center for Disease Control and Prevention, Taizhou, China; ^6^ Department of Chronic Disease Control and Prevention, Haimen City Center for Disease Control and Prevention, Nantong, China; ^7^ School of Epidemiology and Public Health, Faculty of Medicine, University of Ottawa, Ottawa, ON, Canada

**Keywords:** thyroid hormones, puberty, cohort study, iodine sufficient area, female

## Abstract

**Background:**

The onset of puberty is influenced by thyroid function, and thyroid hormones (THs) fluctuate substantially during the period of pubertal development. However, it needs to be further clarified how THs change at specific puberty stages and how it influences pubertal development in girls. So far, longitudinal data from China are scarce.

**Methods:**

A cohort study was conducted among girls during puberty in iodine-sufficient regions of East China between 2017 to 2019. Serum thyroid stimulating hormone (TSH), free triiodothyronine (FT3), and free thyroxine (FT4) were determined for each participant. Thyroid homeostasis structure parameters (THSPs), including the ratio of FT4 to FT3 (FT4/FT3), Jostel’s TSH index (TSHI), and thyroid feedback quantile-based index (TFQI), were calculated. Puberty category scores (PCS), calculated based on the Puberty Development Scale (PDS), was used to assess the stage of puberty. Girls were grouped into three categories according to PCS changes (△PCS) and six categories according puberty stage (B_P_F_P_: pre-pubertal at both baseline and follow-up; B_P_F_L_: pre-pubertal at baseline and late-pubertal at follow-up, respectively; B_P_F_T_: pre-pubertal at baseline and post-pubertal at follow-up, respectively; B_L_F_L_: late-pubertal at both baseline and follow-up; B_L_F_T_: late-pubertal at baseline and post-pubertal at follow-up, respectively; B_T_F_T_: post-pubertal at both baseline and follow-up). Multiple linear regression analyses were used to evaluate the associations of THs changes with pubertal progress.

**Results:**

The levels of serum TSH and FT3 decreased while serum FT4 increased during the study period (P<0.001). In multiple linear regression analyses, after adjustment for covariables, FT3 decreased by an additional 0.24 pmol/L (95% CI: -0.47 to -0.01) in the higher △PCS group than the lower △PCS group. Compared with the B_L_F_L_ group, the B_P_F_T_ group showed an additional decline in FT3 (β= -0.39 pmol/L, 95%CI: -0.73 to -0.04), the B_T_F_T_ group showed a lower decline in TSH (β=0.50 mU/L, 95% CI: 0.21 to 0.80) and a lower decline in TSHI (β=0.24, 95%CI: 0.06 to 0.41), respectively. There was no association of △FT4 or △TFQI with △PCS or the puberty pattern.

**Conclusions:**

Serum TSH and FT3 decreased while serum FT4 increased among girls during puberty. Both the initial stage and the velocity of pubertal development were related to thyroid hormone fluctuations.

## Introduction

Thyroid hormones (THs) play a critical role in physical growth (1), nervous system development (2), body metabolism, and energy expenditure (3). The hypothalamic secretion of the thyrotropin-releasing hormone induces pituitary production of the thyroid stimulating hormone (TSH), which in turn stimulates thyroid follicular cells to produce thyroxine (T4). T4 can be converted to active triiodothyronine (T3) by deiodination in extrathyroidal tissues, and this process is under the control of circulating T4 through negative feedback loops of HPT (4). Free triiodothyronine (FT3) and free thyroxine (FT4) are the free states for T3 and T4 after being released into the blood. TSH, FT3, and FT4 have been considered as the sensitive markers to evaluate thyroid function (5). Thyroid homeostasis structure parameters (THSPs) are often used as the indicators of pituitary thyrotropic function, including Jostel’s TSH index (TSHI) (6), and thyroid feedback quantile-based index (TFQI) (7). The ratio of FT4 to FT3 (FT4/FT3 ratio) may reflect the degree of peripheral T4 to T3 conversion activity (8).

Puberty is a key period from childhood to adulthood with substantial physical and psychological changes (9). In this period, reproductive systems are maturated by hypothalamic-pituitary-gonadal axis (HPG) activation. Evidence suggests that the puberty onset may be influenced by thyroid function (10), and THs fluctuate substantially during the period of pubertal development (11). It is unclear how much THs change for individuals at specific puberty stages or with specific pubertal development patterns. Moreover, studies focusing on the associations between puberty and THs in China are scare and are limited to cross-sectional designs (12).

Thyroid diseases are more likely to occur among females than males in adulthood (13), and epidemiological data have revealed direct effects of estrogen on thyroid function and growth regulation, as well as their potential mechanisms (14). There are inconsistent study results for the gender variations in TSH, FT3, and FT4 levels during puberty. Radicioni et al. found no significant difference in THs between girls and boys during pre-puberty and puberty (15). Marwaha et al. reported that both TSH and FT4 levels decreased with age from 5 to 17 years in girls, but this trend was not observed in boys (16). Zurakowski et al. reported the age-related trend for TSH and FT3 during the period of pubertal development for both sexes, but the trend was more marked in females (17). In this longitudinal study, we aimed to evaluate the associations of THs and THSPs with pubertal development among school girls.

## Methods

### Study Sites and Subject Selection

**Figure 1:**
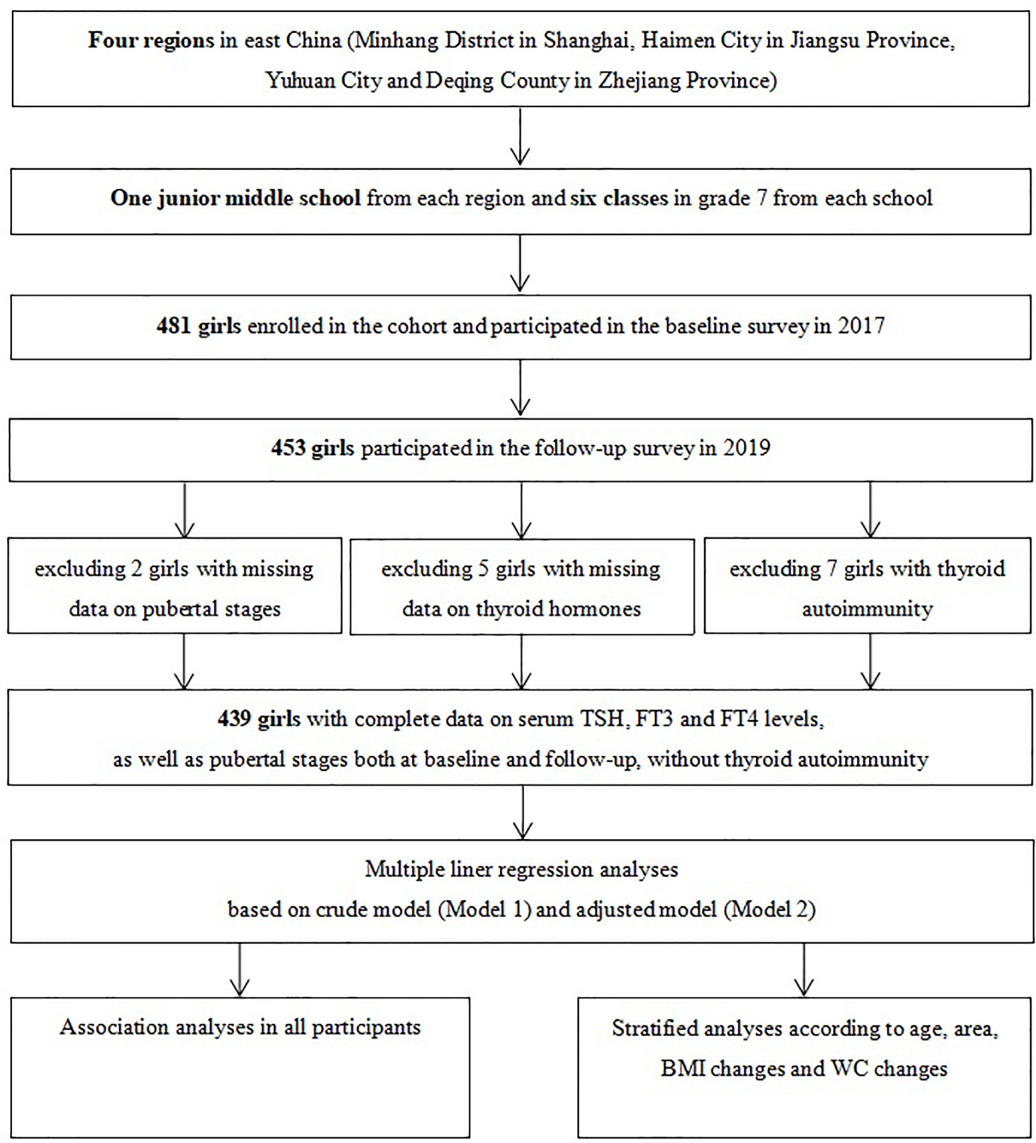
Flow chart for the study.

A purposive sampling method was used to select four iodine-sufficient regions in East China. A description of the study design and sampling method has been detailed in a previous study (18). In brief, one junior middle school was randomly selected from each of the four regions and students were mainly local residents. A total of 481 girls in grade 7 of each school were enrolled into the cohort in October to November 2017 after excluding those with iodine supplements, thyroid disorders, and pituitary abnormalities. Among the 453 girls who participated in the follow-up investigation in 2019, 439 girls had complete data on pubertal stages and thyroid function measures, without any clinical signs or symptoms indicating possible thyroid diseases (**Figure 1**). Informed written consent was obtained from all the participants and their parents, and this study was approved by the ethical review board of the School of Public Health of Fudan University (#2012-03-0350S).

### Anthropometric Measurements

Standing height (cm), weight (kg), and waist circumferences (cm) were measured with participants wearing light clothing and no footwear, using standard protocols. Height and waist circumference (WC) were recorded to the nearest 0.1 cm and weight to the nearest 0.1 kg. Body mass index (BMI) was calculated as weight in kilograms divided by the square of height in meters (kg/m (2)).

### Urine Iodine Content and Thyroid Hormones Determinations

Each participant provided 15-ml spot urine samples in the mornings on Monday and Thursday of a week at both baseline and follow-up for the measurement of urine iodine content (UIC). Approximately 5-ml fasting blood samples were drawn through antecubital vein puncture around 8 am to 9 am. They were immediately centrifuged and the serums were then separated. Samples were kept frozen at -80°C until analysis. Concentrations of thyroid hormones, including TSH, FT3, and FT4 were determined using a chemiluminescent immunoassay (19) following standard methods with proper quality control (ADVIA Centaur CP, Siemens Healthcare Diagnostics Inc, New York, USA).

UIC was determined using the method of inductively coupled plasma mass spectrometry (ICP-MS). ICP-MS method is fast and accurate with easier sample preparation, offering a strong congruence with the S-K method (20). Daily iodine exposure doses were estimated using the formula frequently used for the estimate of daily exposure doses for other similar chemicals (21): DUIO = UIC×DUCrO/(UCr×W) (DUIO: daily urine iodine output, μg; UIC: urine iodine content, μg/L; DUCrO: daily urine creatinine output, mmol; UCr: urine creatinine content, μmol/L; W: weight). Daily urine iodine output (DUIO) and weighted daily urine iodine output (WDUIO) was calculated as follows: (1) DUIO (μg) = UIC×EXP (0.0102×H-0.6854) × 1000 ÷ UCr (log-transformed DUCrO= 0.0102×H−0.6854, H: height) (2) WDUIO (μg) = 2/7 × DUIO on Monday + 5/7 × DUIO on Thursday (18).

### Thyroid Homeostasis Structure Parameters

Three thyroid homeostasis structure parameters (THSPs), including the FT4/FT3 ratio, Jostel’s TSH index (TSHI), and thyroid feedback quantile-based index (TFQI), were calculated by using the following formula: (1) FT4/FT3 ratio = FT4/FT3; (2) TSHI = ln(TSH) + 0.1345 × FT4; and (3) TFQI = (FT4-μ_FT4_)/σ_FT4_-[1-(TSH- μ_TSH_)/σ_TSH_] (7).

### Pubertal Development Assessments

The Puberty Development Scale (PDS) was used to evaluate pubertal status (22). A Puberty Category Score (PCS) was calculated based on three items including menarche (score 0 or 1, namely “without or with”), breast development (score 1 to 5), and body hair growth (score 1 to 4) and five stages (I - V) were defined (Stage I: score = 2 and without menarche; Stage II: score = 3 and without menarche; Stage III: score>3 and without menarche; Stage IV: score ≤ 7 and with menarche; Stage V: scored ≥ 8 and with menarche). Stage I to III were combined as “pre-early-mid-pubertal”, stage IV and stage V were defined as “late-pubertal” and “post-pubertal”, respectively.

Pubertal development patterns were categorized into six groups according to pubertal stage at baseline and follow-up (“B_P_F_P_: pre-pubertal at both baseline and follow-up”, “B_P_F_L_: pre-pubertal at baseline and late-pubertal at follow-up, respectively”, “B_P_F_T_: pre-pubertal at baseline and post-pubertal at follow-up, respectively”, “B_L_F_L_: late-pubertal at both baseline and follow-up”, “B_L_F_T_: late-pubertal at baseline and post-pubertal at follow-up, respectively” and “B_T_F_T_: post-pubertal at both baseline and follow-up”) (12). In addition, girls were also divided into three groups according to the changes in PCS (△ PCS) (“lower: △≤1”, “middle: △ = 2”, “higher: △≥3”).

### Statistical Analysis

The current analysis used data from 439 girls with complete information on pubertal stages and thyroid hormones and excluded those with thyroid autoimmunity. For comparison of baseline characteristics, ANOVA was used for continuous variables, and χ^2^ test and Fisher’s exact test for categorical variables. Wilcoxon matched-pairs signed-ranks test was used to compare the changes in thyroid hormones. Multiple linear regression models were utilized to estimate the associations of THs and THSPs with pubertal development. The statistical significance level was defined as α = 0.05 of two-side probability. All analyses were performed by using the R program (version 4.0.4, R Foundation for Statistical Computing, Vienna, Austria), and all figures were performed using GraphPad Prism software (version 7, GraphPad Prism, California, USA).

## Results

### Characteristics, Thyroid Hormones (THs), and Thyroid Homeostasis Structural Parameters (THSPs)

Information on characteristics, anthropometric indexes, thyroid hormones (THs), and thyroid homeostasis structural parameters (THSPs) of girls is shown in **Tables 1** and **2**. Compared with the 13 to 14-year age group at baseline, younger girls (11 to 12-year age group) were more likely to experience higher △PCS and to be in the B_P_F_P_ or B_P_F_L_ group (*P*<0.001). Waist circumference (WC) increased with age and the changes (△WC) were more evident in girls with higher △PCS or in the B_P_F_P_ or B_P_F_L_ group (*P*<0.001). Serum TSH and FT3 decreased and serum FT4 increased significantly during the study period (*P*<0.001).

**Table 1:** Characteristics, anthropometric indexes, thyroid hormones (THs), and thyroid homeostasis structure parameters (THSPs) of girls with different puberty category score (PCS) changes.

Characteristics	Total	Puberty category scores changes (△PCS)^a^			
		lower (△≤1)	middle (△=2)	higher (△≥3)	*P* value
**No (%)**	439(100)	203(46.24)	107(24.37)	129(29.38)	
**Age at baseline (N,%)**					<0.001
11-12 years	195(44.42)	68(34.87)	48(24.62)	79(40.51)	
13-14 years	244(55.58)	135(55.33)	59(24.18)	50(20.49)	
**Area with different proportions of iodized-salt consumption (N,%)**					0.012
≤90% (Minhang & Yuhuan)	224(51.03)	95(42.41)	49(21.88)	80(35.71)	
>90% (Haimen & Deqing)	215(48.97)	108(50.23)	58(26.98)	49(22.79)	
**BMI (kg/m^2^, Mean ± SD)**					
Baseline (2017)	18.74 ± 3.11	19.55 ± 3.31	18.55 ± 2.95	17.63 ± 2.51	<0.001
Follow-up (2019)	20.05 ± 3.07	20.72 ± 3.30	19.96 ± 2.98	19.06 ± 2.47	<0.001
Changes (△)	1.30 ± 1.65	1.16 ± 1.96	1.44 ± 1.34	1.40 ± 1.34	0.268
**WC (cm, Mean ± SD)**					
Baseline (2017)	65.25 ± 7.74	66.71 ± 7.30	65.96 ± 8.88	62.37 ± 6.58	<0.001
Follow-up (2019)	67.98 ± 7.17	68.91 ± 7.55	67.33 ± 6.60	67.05 ± 6.88	0.041
Changes (△)	2.69 ± 6.35	2.15 ± 5.60	1.43 ± 7.13	4.59 ± 6.40	<0.001
**Weighted daily urine iodine output [µg, Median (P_25_~P_75_)]**					
Baseline (2017)	89.76(57.51~135.04)	78.87(50.37~110.60)	94.81(63.61~136.72)	101.66(71.89~152.06)	<0.001
Follow-up (2019)	47.30(33.30~74.79)	44.33(30.33~63.920)	45.20(32.66~78.70)	57.60(40.33~86.40)	0.001
Changes (△)	-32.02(-73.63~-4.80)	-27.74(-63.43~-1.81)	-36.38(-81.26~-10.41)	-34.52(-89.04~-4.55)	0.248
**TSH [mU/L, Median (P_25_~P_75_)]**					
baseline (2017)	1.90(1.35~2.66)	1.81(1.34~2.64)	1.92(1.35~2.57)	2.01(1.42~2.87)	0.356
follow-up (2019)	1.50(1.05~2.03)	1.54(1.05~2.15)	1.50(1.04~2.00)	1.48(1.09~1.89)	0.589
Changes (△)	-0.42(-0.94~0.08)	-0.28(-0.81~0.16)	-0.37(-1.03~0.10)	-0.56(-1.04~-0.17)	0.024
**FT3 [pmol/L, Median (P_25_~P_75_)]**					
baseline (2017)	5.66(5.27~6.00)	5.44(5.10~5.80)	5.68(5.28~6.03)	5.87(5.55~6.16)	<0.001
follow-up (2019)	4.81(4.47~5.15)	4.73(4.42~5.07)	4.81(4.41~5.17)	4.92(4.64~5.30)	<0.001
Changes (△)	-0.76(-1.10~ -0.45)	-0.71(-1.05~-0.43)	-0.81(-1.18~-0.57)	-0.85(-1.19~-0.42)	0.108
**FT4 [pmol/L, Median (P_25_~P_75_)]**					
baseline (2017)	14.89(13.44~16.51)	15.23(13.86~16.63)	14.94(13.12~16.64)	14.63(13.01~16.05)	0.031
follow-up (2019)	16.50(15.30~17.73)	16.52(15.41~17.69)	16.44(15.26~17.79)	16.57(15.33~17.76)	0.991
Changes (△)	1.57(-0.11~3.14)	1.43(-0.23~2.80)	1.52(-0.18~3.30)	1.80(0.07~3.70)	0.193
**FT4/FT3 [Median (P_25_~P_75_)]**					
baseline (2017)	2.69(2.41~2.99)	2.78(2.51~3.12)	2.7(2.39~2.99)	2.56(2.27~2.78)	<0.001
follow-up (2019)	3.45(3.14~3.75)	3.51(3.21~3.81)	3.45(3.13~3.8)	3.33(3.07~3.59)	0.002
Changes (△)	0.79(0.42~1.08)	0.71(0.39~1.03)	0.8(0.42~1.18)	0.88(0.51~1.17)	0.111
**TSHI [Median (P_25_~P_75_)]**					
baseline (2017)	2.68(2.37~3.01)	2.72(2.37~3.04)	2.63(2.34~3.05)	2.65(2.36~3)	0.863
follow-up (2019)	2.63(2.24~3)	2.65(2.27~3.04)	2.63(2.22~2.98)	2.55(2.21~2.97)	0.542
Changes (△)	-0.06(-0.35~0.28)	-0.06(-0.33~0.26)	-0.06(-0.32~0.23)	-0.05(-0.43~0.31)	0.852
**TFQI [Median (P_25_~P_75_)]**					
baseline (2017)	-0.11(-0.84~0.72)	0.02(-0.75~0.74)	-0.15(-1.03~0.92)	-0.27(-0.92~0.48)	0.393
follow-up (2019)	-0.2(-0.94~0.69)	-0.12(-0.85~0.72)	-0.22(-0.92~0.65)	-0.31(-1.05~0.69)	0.293
Changes (△)	-0.06(-0.91~0.71)	-0.04(-0.86~0.71)	-0.12(-0.95~0.71)	-0.08(-0.96~0.78)	0.977

a PCS changes/△PCS: Changes in puberty category scores (PCS) from baseline to follow-up.

**Table 2:** Characteristics, anthropometric indexes, thyroid hormones (THs), and thyroid homeostasis structure parameters (THSPs) of girls with different puberty patterns.

Characteristics	Total	Puberty pattern^a^					
		B_P_F_P_+B_P_F_L_	B_P_F_T_	B_L_F_L_	B_L_F_T_	B_T_F_T_	*P* value
**No (%)**	439(100)	98(22.32)	62(14.12)	71(16.17)	137(31.22)	71(16.17)	
**Age at baseline (N,%)**							<0.001
11-12 years	195(44.42)	67(34.36)	36(18.46)	30(15.38)	46(23.59)	16(8.21)	
13-14 years	244(55.58)	31(12.70)	26(10.66)	41(16.80)	91(37.30)	55(22.54)	
**Area with different proportions of iodized-salt consumption (N,%)**							<0.001
≤90% (Minhang & Yuhuan)	224(51.03)	64(28.57)	39(17.41)	39(17.41)	56(25.00)	26(11.61)	
>90% (Haimen & Deqing)	215(48.97)	34(15.81)	23(10.70)	32(14.88)	81(37.67)	45(20.93)	
**BMI (kg/m^2^, Mean ± SD)**							
Baseline (2017)	18.74 ± 3.11	16.66 ± 2.12	18.45 ± 2.84	18.06 ± 2.03	19.38 ± 3.22	21.32 ± 2.95	<0.001
Follow-up (2019)	20.05 ± 3.07	18.08 ± 2.07	19.77 ± 2.77	19.35 ± 2.34	20.63 ± 2.91	22.66 ± 3.37	<0.001
Changes (△)	1.30 ± 1.65	1.38 ± 1.19	1.32 ± 1.49	1.28 ± 1.09	1.25 ± 2.27	1.28 ± 1.40	0.983
**WC (cm, Mean ± SD)**							
Baseline (2017)	65.25 ± 7.74	60.16 ± 5.42	64.54 ± 7.73	63.71 ± 4.87	67.26 ± 7.85	70.56 ± 7.91	<0.001
Follow-up (2019)	67.98 ± 7.17	65.11 ± 6.39	68.27 ± 7.35	66.94 ± 6.19	68.84 ± 7.44	71.16 ± 6.93	<0.001
Changes (△)	2.69 ± 6.35	4.85 ± 6.05	3.73 ± 6.62	3.23 ± 5.27	1.58 ± 6.62	0.35 ± 5.90	<0.001
**Weighted daily urine iodine output [µg, Median (P_25_~P_75_)**]							
Baseline (2017)	89.76(57.51~135.04)	92.49(68.86~152.48)	96.49(60.63~145.04)	85.36(55.99~127.58)	87.82(55.84~126.87)	78.62(48.82~119.77)	0.229
Follow-up (2019)	47.30(33.30~74.79)	51.25(34.72~80.28)	62.55(41.76~95.36)	40.01(29.19~64.74)	45.62(32.82~74.96)	44.38(31.57~61.40)	0.004
Changes (△)	-32.02(-73.63~-4.80)	-38.10(-89.20~-8.75)	-29.17(-77.84~5.82)	-35.62(-70.41~-6.68)	-31.45(-71.91~-1.63)	-29.80(-65.38~-2.16)	0.786
**TSH [mU/L, Median (P_25_~P_75_)]**							
baseline (2017)	1.90(1.35~2.66)	2.03(1.42~2.79)	1.89(1.38~2.87)	1.85(1.49~2.67)	1.89(1.30~2.63)	1.79(1.21~2.32)	0.068
follow-up (2019)	1.50(1.05~2.03)	1.52(1.13~2.00)	1.45(0.94~1.80)	1.49(0.98~1.92)	1.50(1.07~2.16)	1.72(1.11~2.34)	0.033
Changes (△)	-0.42(-0.94~0.08)	-0.54-1.04~-0.12)	-0.53(-1.13~-0.24)	-0.57(-1.32~0.08)	-0.27(-0.79~0.12)	-0.21(-0.61~0.37)	0.884
**FT3 [pmol/L, Median (P_25_~P_75_)]**							
baseline (2017)	5.66(5.27~6.00)	5.83(5.52~6.14)	5.93(5.56~6.17)	5.41(5.20~5.74)	5.59(5.10~5.89)	5.40(5.01~5.90)	<0.001
follow-up (2019)	4.81(4.47~5.15)	5.02(4.78~5.37)	4.91(4.63~5.21)	4.66(4.39~4.95)	4.74(4.37~5.09)	4.72(4.42~5.12)	0.024
Changes (△)	-0.76(-1.10~ -0.45)	-0.72(-1.12~-0.29)	-0.95(-1.15~-0.66)	-0.76(-1.06~-0.48)	-0.80(-1.18~-0.49)	-0.69(-0.92~-0.29)	0.048
**FT4 [pmol/L, Median (P_25_~P_75_)]**							
baseline (2017)	14.89(13.44~16.51)	14.38(12.44~15.64)	14.12(12.98~15.97)	15.18(13.85~17.28)	15.20(13.61~16.59)	15.36(13.92~16.78)	0.775
follow-up (2019)	16.50(15.30~17.73)	16.35(15.06~17.97)	16.63(15.51~17.84)	16.73(15.53~17.65)	16.47(15.46~17.58)	16.20(15.04~17.48)	0.409
Changes (△)	1.57(-0.11~3.14)	2.06(0.46~3.73)	2.36(0.51~3.78)	1.32(-0.39~2.90)	1.11(-0.26~3.09)	0.58(-0.50~2.60)	0.448
**FT4/FT3 [Median (P_25_~P_75_)]**							
baseline (2017)	2.69(2.41~2.99)	2.49(2.2~2.73)	2.45(2.26~2.78)	2.81(2.51~3.14)	2.74(2.49~3.06)	2.86(2.58~3.17)	<0.001
follow-up (2019)	3.45(3.14~3.75)	3.27(2.94~3.56)	3.36(3.08~3.64)	3.6(3.21~3.87)	3.49(3.24~3.78)	3.44(3.18~3.79)	<0.001
Changes (△)	0.79(0.42~1.08)	0.8(0.53~1.11)	1(0.56~1.31)	0.75(0.38~1.03)	0.84(0.41~1.07)	0.67(0.23~0.94)	0.139
**TFQI [Median (P_25_~P_75_)]**							
baseline (2017)	2.68(2.37~3.01)	2.61(2.34~3.02)	2.62(2.21~2.95)	2.76(2.45~3.06)	2.66(2.4~3.13)	2.61(2.27~2.96)	0.309
follow-up (2019)	2.63(2.24~3)	2.6(2.25~3)	2.52(2.14~2.94)	2.62(2.25~2.91)	2.65(2.27~3.05)	2.73(2.31~3.04)	0.565
Changes (△)	-0.06(-0.35~0.28)	-0.05(-0.31~0.35)	-0.03(-0.5~0.31)	-0.19(-0.41~0.1)	-0.02(-0.36~0.21)	0(-0.29~0.37)	0.203
**TFQI [Median (P_25_~P_75_)]**							
baseline (2017)	-0.11(-0.84~0.72)	-0.36(-0.94~0.41)	-0.31(-1.19~0.43)	0.26(-0.64~0.8)	0(-0.62~0.98)	0(-0.88~0.67)	0.020
follow-up (2019)	-0.2(-0.94~0.69)	-0.22(-1.02~0.72)	-0.36(-1.12~0.67)	-0.15(-0.83~0.64)	-0.17(-0.8~0.69)	-0.13(-0.85~0.82)	0.750
Changes (△)	-0.06(-0.91~0.71)	-0.02(-0.66~1.14)	0.09(-0.88~1.06)	-0.29(-1.03~0.43)	-0.15(-1~0.69)	0.04(-0.79~0.84)	0.139

a Puberty pattern: “B_P_F_P_+B_P_F_L_”, pre-pubertal at both baseline and follow-up & pre-pubertal at baseline and late-pubertal at follow-up, respectively; “B_P_F_T_, pre-pubertal at baseline and post-pubertal at follow-up, respectively”, “B_L_F_L_, late-pubertal at both baseline and follow-up”, “B_L_F_T_, late-pubertal at baseline and post-pubertal at follow-up, respectively” and “B_T_F_T_, post-pubertal at both baseline and follow-up”.

### Changes in Thyroid Hormones (△THs) and Thyroid Homeostasis Structural Parameters (△THSPs) and Their Associations With Puberty Category Scores (△PCS) and Puberty Pattern

In multiple linear regression analyses, after adjustment for covariables (Model 2), FT3 decreased additional 0.24 pmol/L (95% CI: -0.47 to -0.01) in the higher △PCS group than the lower △PCS group. Compared with the B_L_F_L_ group, the B_P_F_T_ group showed additional decline in FT3 (β = -0.39 pmol/L, 95%CI: -0.73 to -0.04), the B_T_F_T_ group showed lower decline in TSH (β = 0.50 mU/L, 95% CI: 0.21 to 0.80)and lower decline in TSHI (β = 0.24, 95% CI: 0.06 to 0.41), respectively. There was no association of △FT4 or △TFQI with △PCS or puberty pattern (**Tables 3**, **4**).

**Table 3:** β and 95% confidence interval (95% CI) for thyroid hormones changes (△THs) according to puberty development by multiple liner regression analyses.

Group	N(%)^a^	*Median* (P_25_~P_75_)	β (95% CI)	
			Model 1^d^	Model 2^e^
**△TSH (mU/L)**				
PCS changes ^b^				
lower (△≤1)	203(46.24)	-0.28(-0.81~0.16)	0.00	0.00
middle (△=2)	107(24.37)	-0.37(-1.03~0.10)	-0.08(-0.30~0.14)	-0.09(-0.30~0.13)
higher (△≥3)	129(29.38)	-0.56(-1.04~ -0.17)	-0.25(-0.45~ -0.04)^*^	-0.14(-0.35~0.07)
Per-1 SD	–	–	-0.09(-0.19~0.01)	-0.05(-0.15~0.05)
Puberty pattern ^c^				
B_P_F_P_+B_P_F_L_	98(22.32)	-0.54(-1.04~ -0.12)	0.09(-0.19~0.37)	0.19(-0.10~0.47)
B_P_F_T_	62(14.12)	-0.53(-1.13~ -0.24)	0.02(-0.30~0.33)	0.07(-0.23~0.38)
B_L_F_L_	71(16.17)	-0.57(-1.32~0.08)	0.00	0.00
B_L_F_T_	137(31.22)	-0.27(-0.79~0.12)	0.28(0.01~0.54)^*^	0.24(-0.01~0.50)
B_T_F_T_	71(16.17)	-0.21(-0.61~0.37)	0.58(0.28~0.88)^***^	0.50(0.21~0.80)^**^
**△FT3 (pmol/L)**				
PCS changes ^b^				
lower (△≤1)	203(46.24)	-0.71(-1.05~ -0.43)	0.00	0.00
middle (△=2)	107(24.37)	-0.81(-1.18~ -0.57)	-0.14(-0.37~0.10)	-0.14(-0.38~0.10)
higher (△≥3)	129(29.38)	-0.85(-1.19~ -0.42)	-0.19(-0.41~0.03)	-0.24(-0.47~ -0.01)^*^
Per-1 SD	–	–	-0.07(-0.18~0.03)	-0.10(-0.21~0.01)
Puberty pattern ^c^				
B_P_F_P_+B_P_F_L_	98(22.32)	-0.72(-1.12~ -0.29)	-0.21(-0.52~0.09)	-0.26(-0.57~0.06)
B_P_F_T_	62(14.12)	-0.95(-1.15~ -0.66)	-0.36(-0.70~ -0.02)^*^	-0.39(-0.73~ -0.04)^*^
B_L_F_L_	71(16.17)	-0.76(-1.06~ -0.48)	0.00	0.00
B_L_F_T_	137(31.21)	-0.80(-1.18~ -0.49)	-0.27(-0.56~0.01)	-0.26(-0.55~0.03)
B_T_F_T_	71(16.17)	-0.69(-0.92~ -0.29)	-0.12(-0.45~0.21)	-0.10(-0.42~0.24)
**△FT4 (pmol/L)**				
PCS changes ^b^				
lower (△≤1)	203(46.24)	1.43(-0.23~2.80)	0.00	0.00
middle (△=2)	107(24.37)	1.52(-0.18~3.30)	0.04(-0.58~0.67)	0.07(-0.51~0.67)
higher (△≥3)	129(29.38)	1.80(0.07~3.70)	0.41(-0.18~1.00)	-0.17(-0.74~0.41)
Per-1 SD	–	–	0.20(-0.08~0.49)	-0.03(0.14~ -0.31)
Puberty pattern ^c^				
B_P_F_P_+B_P_F_L_	98(22.32)	2.06(0.46~3.73)	0.47(-0.34~1.27)	0.00(-0.78~0.77)
B_P_F_T_	62(14.12)	2.36(0.51~3.78)	0.52(-0.38~1.42)	0.27(-0.58~1.12)
B_L_F_L_	71(16.17)	1.32(-0.39~2.90)	0.00	0.00
B_L_F_T_	137(31.21)	1.11(-0.26~3.09)	-0.42(-1.17~0.34)	-0.20(-0.91~0.51)
B_T_F_T_	71(16.17)	0.58(-0.50~2.60)	-0.63(-1.50~0.24)	-0.18(-1.01~0.64)

a N(%): The number and proportions of girls with different characteristics;

b PCS changes: Changes in puberty category scores (PCS) from baseline to follow-up;

c Puberty pattern: “B_P_F_P_+B_P_F_L_”, pre-pubertal at both baseline and follow-up & pre-pubertal at baseline and late-pubertal at follow-up, respectively; “B_P_F_T_, pre-pubertal at baseline and post-pubertal at follow-up, respectively”, “B_L_F_L_, late-pubertal at both baseline and follow-up”, “B_L_F_T_, late-pubertal at baseline and post-pubertal at follow-up, respectively” and “B_T_F_T_, post-pubertal at both baseline and follow-up”;

d Model 1: Crude β(95% CI);

e Model 2: Adjusted β(95% CI) after age at baseline, area, △BMI, and △WC.

^*^P < 0.05; ^**^P < 0.01. ^***^P < 0.001.

**Table 4:** β and 95% confidence interval (95% CI) for changes in thyroid homeostasis structure parameters (△ THSPs) according to puberty development by multiple liner regression analyses.

Group	N(%)^a^	*Median* (P_25_~P_75_)	β (95% CI)	
			Model 1^d^	Model 2^e^
**△FT4/FT3**				
PCS changes ^b^				
lower (△≤1)	203(46.24)	0.71(0.39~1.03)	0.00	0.00
middle (△=2)	107(24.37)	0.8(0.42~1.18)	0.05(0, 0.18)	0.07(-0.07, 0.20)
higher (△≥3)	129(29.38)	0.88(0.51~1.17)	0.09(-0.04, 0.21)	0.03(-0.09, 0.16)
Per-1 SD	–	–	0.03(-0.03, 0.09)	0.04(-0.02, 0.10) 4()
Puberty pattern ^c^				
B_P_F_P_+B_P_F_L_	98(22.32)	0.8(0.53~1.11)	0.05(-0.12, 0.22)	0.01(-0.17, 0.18)
B_P_F_T_	62(14.12)	1(0.56~1.31)	0.17(-0.02, 0.36)	0.15(-0.03, 0.34)
B_L_F_L_	71(16.17)	0.75(0.38~1.03)	0.00	0.00
B_L_F_T_	137(31.22)	0.84(0.41~1.07)	0.02(-0.14, 0.18)	0.04(-0.11, 0.21)
B_T_F_T_	71(16.17)	0.67(0.23~0.94)	-0.1(-0.29, 0.08)	-0.04(-0.23, 0.14)
**△TSHI**				
PCS changes ^b^				
lower (△≤1)	203(46.24)	-0.06(-0.33~0.26)	0.00	0.00
middle (△=2)	107(24.37)	-0.06(-0.32~0.23)	-0.01(-0.13, 0.12)	-0.01(-0.14, 0.11)
higher (△≥3)	129(29.38)	-0.05(-0.43~0.31)	-0.02(-0.14, 0.09)	-0.06(-0.19, 0.06)
Per-1 SD	–	–	0.01(-0.05, 0.07)	-0.02(-0.07, 0.04)
Puberty pattern ^c^				
B_P_F_P_+B_P_F_L_	98(22.32)	-0.05(-0.31~0.35)	0.15(-0.01, 0.31)	0.11(-0.05, 0.27)
B_P_F_T_	62(14.12)	-0.03(-0.5~0.31)	0.09(-0.09, 0.26)	0.07(-0.11, 0.25)
B_L_F_L_	71(16.17)	-0.19(-0.41~0.1)	0.00	0.00
B_L_F_T_	137(31.21)	-0.02(-0.36~0.21)	0.06(-0.08, 0.21)	0.08(-0.07, 0.23)
B_T_F_T_	71(16.17)	0(-0.29~0.37)	0.21(0.04, 0.38)^*^	0.24(0.06, 0.41)^**^
**△TFQI**				
PCS changes ^b^				
lower (△≤1)	203(46.24)	-0.04(-0.86~0.71)	0.00	0.00
middle (△=2)	107(24.37)	-0.12(-0.95~0.71)	-0.08(-0.39, 0.23)	-0.07(-0.38, 0.24)
higher (△≥3)	129(29.38)	-0.08(-0.96~0.78)	-0.05(-0.34, 0.24)	-0.22(-0.53, 0.08)
Per-1 SD	–	–	0.01(-0.14, 0.15)	-0.06(-0.20, 0.09)
Puberty pattern ^c^				
B_P_F_P_+B_P_F_L_	98(22.32)	-0.02(-0.66~1.14)	0.31(-0.09, 0.71)	0.17(-0.24, 0.58)
B_P_F_T_	62(14.12)	0.09(-0.88~1.06)	0.23(-0.21, 0.68)	0.17(-0.28, 0.62)
B_L_F_L_	71(16.17)	-0.29(-1.03~0.43)	0.00	0.00
B_L_F_T_	137(31.21)	-0.15(-1~0.69)	0.08(-0.3, 0.45)	0.15(-0.23, 0.52)
B_T_F_T_	71(16.17)	0.04(-0.79~0.84)	0.25(-0.18, 0.68)	0.39(-0.04, 0.82)

a N(%): The number and proportions of girls with different characteristics;

b PCS changes: Changes in puberty category scores (PCS) from baseline to follow-up;

c Puberty pattern: “B_P_F_P_+B_P_F_L_”, pre-pubertal at both baseline and follow-up & pre-pubertal at baseline and late-pubertal at follow-up, respectively; “B_P_F_T_, pre-pubertal at baseline and post-pubertal at follow-up, respectively”, “B_L_F_L_, late-pubertal at both baseline and follow-up”, “B_L_F_T_, late-pubertal at baseline and post-pubertal at follow-up, respectively” and “B_T_F_T_, post-pubertal at both baseline and follow-up”;

d Model 1: Crude β(95% CI);

e Model 2: Adjusted β(95% CI) after age at baseline, area, △BMI, and △WC.

^*^P < 0.05; ^**^P < 0.01.

**Figure 2:**
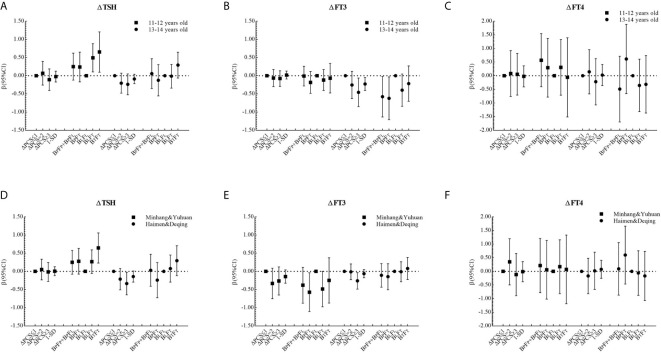
β and 95% confidence interval (95% CI) for ΔTHs according to ΔPCS and puberty pattern by multiple liner regression analyses (Model 2) after being stratified by age at baseline or area with different proportions of iodized-salt consumption.

**Figure 3:**
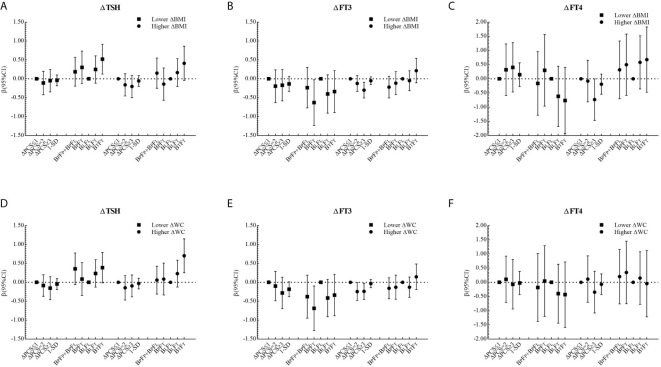
β and 95% confidence interval (95% CI) for ΔTHs according to ΔPCS and puberty pattern by multiple liner regression analyses (Model 2) after being stratified by ΔBMI level or ΔWC level.

The corresponding βs and 95% CIs in stratified analyses are depicted in **Figures 2** and **3**. Higher △TSH was observed in the B_L_F_T_ group, especially in younger girls. △FT3 was negatively associated with △PCS in girls aged 13 to 14 years at the baseline, those from Haimen or Deqing, as well as those with higher △BMI or higher △WC.

## Discussion

In this cohort study, we found that serum TSH and FT3 declined while serum FT4 was elevated among girls during the study period, and those changes were pronounced among those undergoing faster puberty progress (higher △PCS). Girls in the pre-early-mid-pubertal stage or primary stage at baseline and in post-pubertal stage or advanced stage at follow-up (B_P_F_T_), experienced larger changes in THs compared with those in the completed pubertal category (B_T_F_T_).

The puberty-dependent differences in TSH, FT3, and FT4 observed in our study were consistent with the results from most previous cross-sectional data (15, 23, 24), which suggests a complicated interplay between the hypothalamic-pituitary-gonadal axis (HPG) and the hypothalamic-pituitary-thyroid axis (HPT) during pubertal development. HPG is active in the embryonic and early postnatal stages of life and is subsequently restrained during childhood, and its reactivation culminates in puberty initiation (25). Menarche is one of the landmarks of pubertal events, representing the development of the endometrium stimulated by a surge of estrogen (26). Testosterone stimulates the expression of insulin-like growth factor 1 (IGF-1) and sodium iodide symporter (NIS) genes in thyrocytes of both sexes, while estrogen stimulates them only in females (27). Estrogen may regulate the thyroid function metabolism and promote thyrocytes proliferation by mediating the binding on the estrogen receptor (ER), the soluble intracellular nuclear receptors (14).

Our study indicates that both stage and velocity of pubertal development relate to the fluctuations in THs. We considered girls in late-pubertal stage at both baseline and follow-up (B_L_F_L_) as the reference group who had experienced menarche with the same pubertal stage during the study period. Compared with the B_L_F_L_ group, girls with initial puberty and without menarche at the beginning of the study (B_P_F_L_ and B_P_F_T_) showed larger △THs and △THSPs, whereas those with advanced puberty and with menarche at the beginning of the study (B_L_F_T_ and B_T_F_T_) experienced smaller changes. Similarly, girls with faster pubertal progress (higher △PCS) correlated with larger △THs. The onset of menarche for these girls occurred in the study period. Previous studies suggested that reference ranges for thyroid hormones in children and adolescents were substantially wider than ones in adults (15, 28, 29). A pre-pubertal surge in TSH, followed by a transient rise in circulating T3 and T4, along with an enhanced peripheral conversion of T4 to T3, was reported in 9-year-old children, while a decreasing or constant TSH, alongside a progressive decline in T3 and T4 was reported at increasing maturation of puberty (30, 31). In addition, the fluctuations in THs might represent the elevated activities in the thyroid gland preparing for pubertal development, or an outcome of fluctuations in other factors including growth hormone (GH), insulin-like growth factor 1 (IGF-1), and basal luteinizing hormone (LH) (11, 32–34).

The strengths of our study include the longitudinal study design in iodine-sufficient regions, the relatively comprehensive indicators for thyroid function, and the objective assessment of pubertal progress for each girl. Our study also had limitations. First, assessments of pubertal stage were confined to broader categories (pre-early-mid-pubertal/late-pubertal/post-pubertal) due to the limited sample size. Second, the participants were predominantly selected from iodine-sufficient regions in East China, and the findings may not be fully generalized to other populations. Third, there was a lack of information on thyroxine-binding globulin (TBG) and deiodinase, and thyroid’s secretory capacity (SPINA-GT) or total deiodase activity (SPINA-GD) could be calculated.

## Conclusion

In this longitudinal study, we found that serum TSH and FT3 declined while serum FT4 was elevated among girls during puberty. Both the stage and the velocity of pubertal development were related to thyroid hormone fluctuations.

## Data Availability Statement

The datasets for this article are not publicly available because: [personal information of participants]. Requests to access the datasets should be directed to [WN, na.wang@fudan.edu.cn].

## Ethics Statement

Informed written consents were obtained from all the participants and their parents, and this study was approved by the ethical review board of School of Public Health of Fudan University (#2012-03-0350S).

## Author Contributions

DH, XD, MS, PH, NW, YC, and QJ contributed to the study design. YW, DH, CF, FJ, QX, and NW contributed to data acquisition and collection. YW, NW, and YC contributed to data analysis and interpretation. YW and NW drafted the manuscript. All authors contributed to the article and approved the submitted version.

## Funding

This work was supported by grants from the National Natural Science Foundation of China (Grant No. 81602806), the Shanghai Leading Academic Discipline Project of Public Health (Grant No.15GWZK0801), the Minhang District Natural Science Research Project (Grant No.2018MHZ010), and the Fudan-Minhang Health Complex Project (Grant No.2019FM07).

## Conflict of Interest

The authors declare that the research was conducted in the absence of any commercial or financial relationships that could be construed as a potential conflict of interest.
